# Differential Pressure Spirometry for Mechanical Ventilation Using Dichotomic Search

**DOI:** 10.1109/TIM.2021.3116307

**Published:** 2021-09-29

**Authors:** Noé A. Rodríguez-Olivares, Luciano Nava-Balanzar, Leonardo Barriga-Rodríguez

**Affiliations:** Division of Electrical Engineering and ElectronicsCenter for Engineering and Industrial Development (CIDESI) Queretaro 76125 Mexico; School of EngineeringAnahuac University Queretaro 76246 Mexico; Department of EngineeringLatin American Technological University (UTEL) Naucalpan de Juarez Estado de Mexico 53370 Mexico

**Keywords:** Binary search algorithm, COVID-19, dichotomic search, D-lite, FiO₂, flow estimation, mechanical ventilation, spirometry, tidal volume, venturi

## Abstract

In invasive mechanical ventilation (IMV), it is critical that the flow value is estimated correctly, as it is used as a trigger variable for ventilatory assistance. Furthermore, the numerical integration of the flow allows the calculation of the total volume per breath (tidal volume), which clinicians use to identify trauma or lung capacity in the patient. The current COVID-19 pandemic has demonstrated the need to develop safe and efficient techniques for measuring this spirometry variable because many mechanical ventilators delivered to hospitals were unable to measure it directly. A good device to estimate flow is a D-lite sensor, which works by the Venturi effect, is cheap, reusable, and proximal to the patient. However, the regressions applied to the flow estimation model are limited for use in real conditions. This article presents a flow estimation method that uses a D-Lite device, a fraction of inspired oxygen (FiO_2_) cell, and two pressure sensors as critical items. Our novel method adapts the dichotomous search algorithm instead of conventional regression algorithms to estimate flow using a D-lite sensor; this change in the standard procedure allowed us a fast calibration process, a good low-flow estimation, and low computational time for flow estimation. The method was validated experimentally to compute the tidal volume according to the measurement requirement error range of +/−10%. The consideration of FiO_2_ percentage in the gas mixture and the good low-flow estimation make this novel method useful for real ventilation conditions. The flow calculations have been performed at different ambient conditions and compared with gas analyzers show an average relative error of up to 4.86%. Finally, we present an analysis of the error flow estimation considering the variation in each variable. Technical recommendations for applying this novel method to achieve IMV safely are presented, based on the capabilities of the embedded system used by developers.

## I. Introduction

Mechanical ventilation is an artificial respiration procedure that uses a mechanical device to fully or partially supply ventilatory function to a person. A mechanical ventilator is a medical system capable of generating positive pressure for an air and oxygen gas mixture so that a pressure gradient appears between its pneumatic circuit and the patient. It is important to clarify that mechanical ventilation is not a treatment in itself, but is a life support technique that maintains respiratory function while clinical staff perform other treatments or clinical procedures on patients [1].

Endotracheal intubation is when clinical places a plastic tube (polyvinyl) in the trachea of a patient to provide artificial mechanical ventilation [2]. This intubation is required for multiple reasons [3]–[6], for example: 1) for postoperative treatments; 2) when there is a high risk of regurgitation of gastric contents and aspiration into the respiratory tract causing obstructive pulmonary disease; 3) in cardio-respiratory arrest where a low level of consciousness can trigger a prolonged apnea (caused by poisoning, trauma); and 4) acute respiratory diseases such as influenza and COVID-19, which cause acute respiratory failure [7]. For the latter cases, the duration of these illnesses can last from days up to weeks, and mechanical ventilation may be required for more than a week [8]. One of the most dangerous outcomes of these infections is acute respiratory distress syndrome (ARDS), which is characterized by inflammation of the lungs that leads to impaired gas exchange [9].

During mechanical ventilation, the ventilator injects a gas mixture (air plus oxygen) in a controlled way. The three primary variables monitored by the mechanical ventilator during the gas injection are airway pressure, flow, and the tidal volume (numerical integration of the flow) [10]. Processing these variables, as well as other critical ventilatory parameters such as positive end-expiratory pressure (PEEP), FiO_2_, and plateau pressure allow the clinical treatment evaluation [11]–[16]. Airway pressure can be measured easily by applying the Pascal principle. However, the flow measurement presents a significant challenge because the device must be directly connected to the patient airway. Using inadequate instrumentation for the flow measurement can introduce pathogens and solids into the patient’s airway and generate severe clinical problems.

The COVID-19 pandemic generated a growing demand for mechanical ventilators [17]. Despite vaccinations being underway [18], the virus is still present, another flu virus risk is latent, and several economies cannot afford to buy first-world ventilators. Due to the complexity of measuring flow, some ventilators produced were only capable of measuring pressure [19]–[21]. Some ventilators [22]–[24] operate by gradually tightening the angle of an artificial manual breathing unit (AMBU), which allows them to estimate the volume. Problems with these systems can occur when the ABMU bag does not return to its initial position, which reduces the volume delivered to the patient as a result of the tightened angle. In addition, these systems do not monitor the flow delivered to the patient, and therefore insufflation is not ensured, which is very risky from a medical perspective. Other ventilators [25] use flow sensors considered expensive, which is a barrier to their continued use in intensive care units (ICUs). The sensors were also not available during the pandemic due to their long manufacturing time. Another solution to estimate flow is to use the Bernoulli principle [26]; however, the flow must be measured proximal to the patient to ensure the correct tidal volume, otherwise, it is necessary to take account of any leaks present in the system to ensure that the flow in the circuit is being insufflated to the patient.

A good alternative to measure flow is to use a Venturi device [27], which is cheap, proximal to the patient, reusable, can be 3-D printed, and allows the estimation of the flow via the Venturi effect. Some ventilators [28], [29] already use this device; however, they use a numerical regression to linearize the model and obtain only one proportional constant $k$ to use in all flow ranges; this procedure generates a high error for low-flow values. The high error for low-flow measurements is not desirable because the flow can be used as a trigger (in an interval from 1 to 20 L/min) for assistant ventilatory cycles [30]. Additionally, ventilation in volume-control mode (where the flow is controlled) could be dangerous to the patient, causing volutrauma or acteletrauma [31]. One option is to estimate all model variables [27]; however, this process can significantly lengthen the response time for the development of measurement systems for ventilators during pandemics. The alternative method for flow estimation that we proposed to resolve the problem with low flow values was to eliminate the regression step and store a dataset, in order to apply a binary search algorithm, which works with local instead of global errors. In this way, our novelty is the adaptation of the dichotomic search to this specific application. It should be noted that the impact on gas density modification caused by air and oxygen mixture has not been analyzed. This consideration is beneficial because the FiO_2_ value is varied during connection with the patient according to their clinical evaluation [32]. All the points previously mentioned are essential when using mechanical ventilators for emergencies such as ARDS, where assistant ventilation with high oxygen percentages is required.

The major contribution of this article is described as follows. We proposed a novel method for clinical flow estimation with a fast calibration time, low computation time, and reasonable accuracy even for low-flow values, with total errors for inspired, and expired volume within the margin of 10% [33], [34]. The application is in the medical field, for mechanical ventilation systems. In this method, we first proposed a calibration process that reduces the error in approximating model parameters. Later, during flow estimation, a dichotomous search algorithm (binary search) is applied instead of numerical regression. The proposed estimation method has good precision and does not require expensive equipment, making it suitable for in-field calibration and fast step production.

The remainder of this article is organized as follows. Section II introduces the theory of the D-Lite spirometer, including the five-parameter model. In Section III, the material and experimental setup are introduced. In Section IV, the experimental results using the method are carried out. In Section V, we discuss the accuracy and uncertainty of the proposed method, and present technical recommendations for applying this novel method based on the capabilities of the embedded system used. In Section VI, the study is summarized.

## II. Theory

**Fig. 1.**
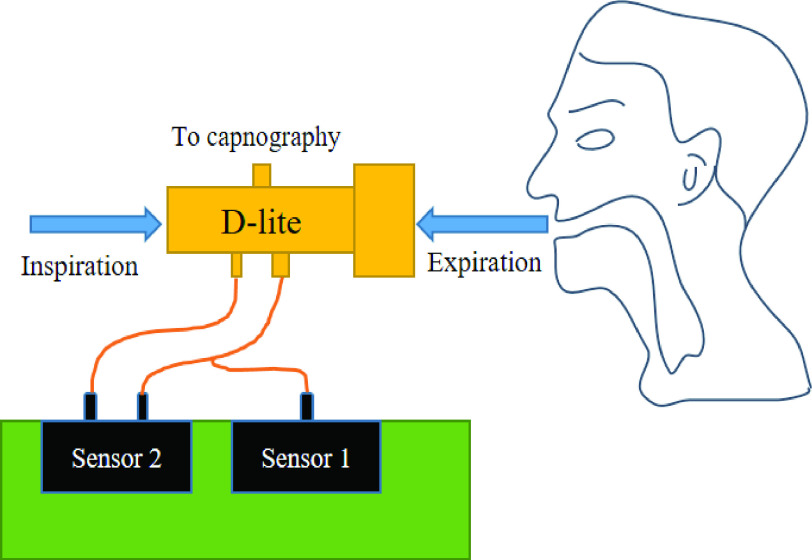
Connection diagram for D-lite spirometer, the capnography port leaves the connection to vital signs monitor. With the other two ports, their differential pressure allows estimating the flow (sensor 2). Sensor 1 obtains the airway pressure.

The D-Lite spirometer piece incorporates flow, airway pressure, and sidestream gas measurement. This spirometer was designed for reliable and accurate results, with continuous monitoring even when exposed to humidity and mucus [27]. The device has three ports; one is for capnography monitoring. The other two ports are used to monitor pressure and flow, which is estimated by measuring the differential pressure between the two sensor ports, as shown in Fig. 1. Pressure sensor number 1 measures the relative pressure, which is the airway pressure compared to ambient pressure and should range from 0- to 100-cm H_2_O. Differential pressure sensor (DPS) number 2 measures the differential pressure in a preferred range of −3- to 3-cm H_2_O, obtained between the two ports to estimate the flow value. The flow rate is estimated by applying the Venturi effect in both directions, inspiration, and expiration, using the following equation:

\begin{equation*} Q=C_{\text {d}} A_{\text {c}} \sqrt {\frac {2 \Delta P}{\rho }} \tag{1}\end{equation*}

where $Q$ is the gas flow in L/min, $C_{\mathrm{ d}}$ is the discharge coefficient factor, $A_{\mathrm{ c}}$ is the total cross-sectional area of the three equal spirometer ports in mm^2^, $\Delta P$ is the differential pressure between the two ports from D-Lite spirometer in cm H_2_O, and $\rho $ is the density of the gas flowing in the spirometer in g/cm^3^ [27].

Some important features of this model are as follows; due to the quadratic relationship between flow $Q$ and differential pressure $\Delta P$, the estimation of low flow values is considered complicated [27], [35]. The discharge coefficient $C_{\mathrm{ d}}$ is a function of the Reynolds number and the relationship between the area through which the flow $Q$ passes and the area obstructed by the ports. This coefficient value considers the gas viscosity and the tube diameter. $C_{\mathrm{ d}}$ was 0.75 at a flow value of 7 to 8 L/min in the first D-Lite proposed by Meriläinen *et al.* [27] and its value decreased to 0.65 when the flow was increased or decreased by 0.1 L/min.

## III. Material and Experimental Setup

### A. Experimental Setup

**Fig. 2.**
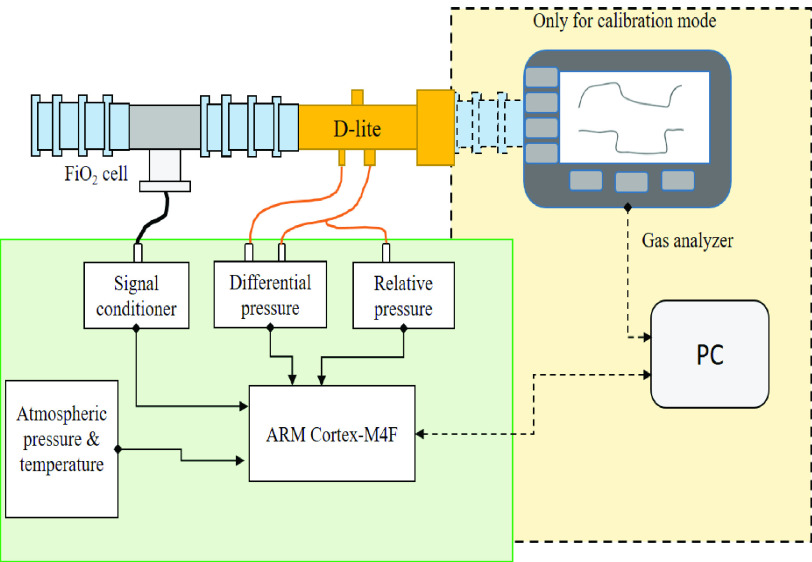
Complete system required for estimating flow, with pressure and temperature sensors, considering the fraction of inspired oxygen (FiO_2_). In dotted lines, the connections are necessary for calibration mode, where the gas analyzer sends the flow ( $Q$) value to the computer.

**Fig. 3.**
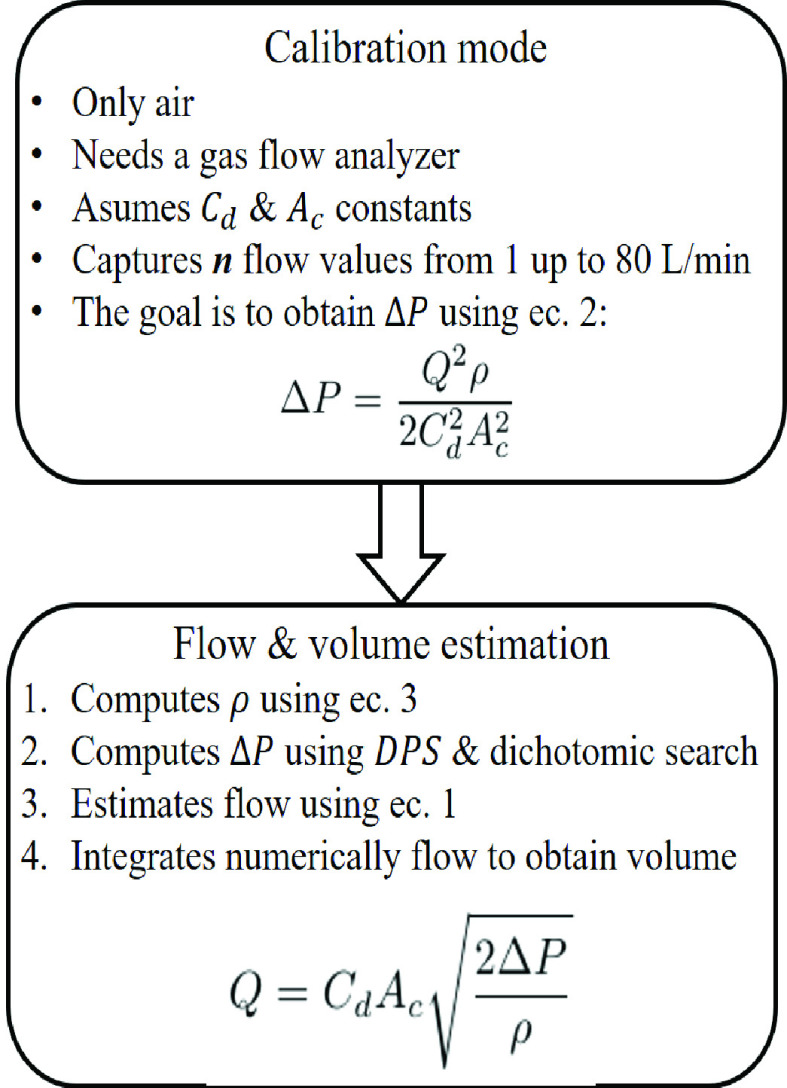
Block diagram of the proposed method.

For the development of this method, we used a D-lite spirometer for the ventilatory measurement of the patient. A fraction of inspired oxygen (FiO_2_) cell was used to estimate the percentage of oxygen (O_2_) insufflated and involved in gas exchange. Equipment used included an ARM Cortex-M4F processor TM4C123GXL with a 32-b CPU for computation (Texas Instruments, Dallas, TX, USA), atmospheric pressure and temperature sensor MS5607-02BA03 for density estimation (MEAS, Hampton, Switzerland), a DPS SDP32-125PA-TR (Sensirion, Stäfa, Switzerland) for estimating flow, and a relative pressure sensor HSCMRRN005PDSA5 (Honeywell, Morris Plains, NJ, USA) for airway pressure. We selected these pressure sensors for their accuracy and offset stability. Fig. 2 shows the connection between the different elements of the system. Fig. 3 shows the block diagram of the proposed method. The VT900A (Fluke, Everett, WA, USA) gas analyzer was also used for the calibration step and method validation. We used two lungs model type SmartLung Adult 1L each one with applicable volume from 0 to 600 mL (IMT Analytics, Buchs, Switzerland). For air supply in the experimental results, we used a similar prototype to [22], [23].

### B. Proposed Calibration Method

The main goal of the calibration process is to obtain the theoretical differential pressure value $\Delta P$ which should be measured between the two ports of the D-lite spirometer sensor number 2 (see Fig. 1). To achieve this, we rearranged (1) to arrive at (2) (see Fig. 3). The gas analyzer connection in calibration mode (see Fig. 2) is needed to obtain the instantaneous flow value $Q$, computing the gas density $\rho $ and considering the values of $C_{\mathrm{ d}}= 0.7$ and $A_{\mathrm{ c}}= 58$ mm^2^ constants. However, if the estimated value $\Delta P$ does not coincide with the measured value, the relationship between $\Delta P$ and the differential pressure value (from sensor 2 Fig. 1) is the proportional factor of adjustment that must be considered for each flow value. This intrinsic factor can correspond to $C_{\mathrm{ d}}$ variation or an intrinsic adjustment if there is a change in the $A_{\mathrm{ c}}$ parameter, which allows using this method for spirometers of another size just by following the same calibration process:

\begin{equation*} \Delta P=\frac {Q^{2} \rho }{2 C_{\text {d}}^{2} A_{\text {c}}^{2}}. \tag{2}\end{equation*}

For the calibration step (see Fig. 2), we followed the recommendations of Meriläinen *et al.* [27]. The system is connected to an air-only source that can be regulated manually or automatically to obtain the estimated value $\Delta P$ and its corresponding value from the measured DPS. The airflow is regulated and captured in intervals of approximately 1 L/min, starting from 1 and up to 40 L/min; subsequently, then increasing by intervals of 5 L/min until reaching the sensor saturation point, which is around 80 L/min in our case. The data capture occurs in intervals of 1 L/min in the range of 1–40 L/min to reduce the error reported for low-flow estimation [27], [35]. Flow values between 1 and 20 L/min are used as triggers in ventilatory assistance; therefore, having a reasonable estimation of these values is very important.

**Fig. 4.**
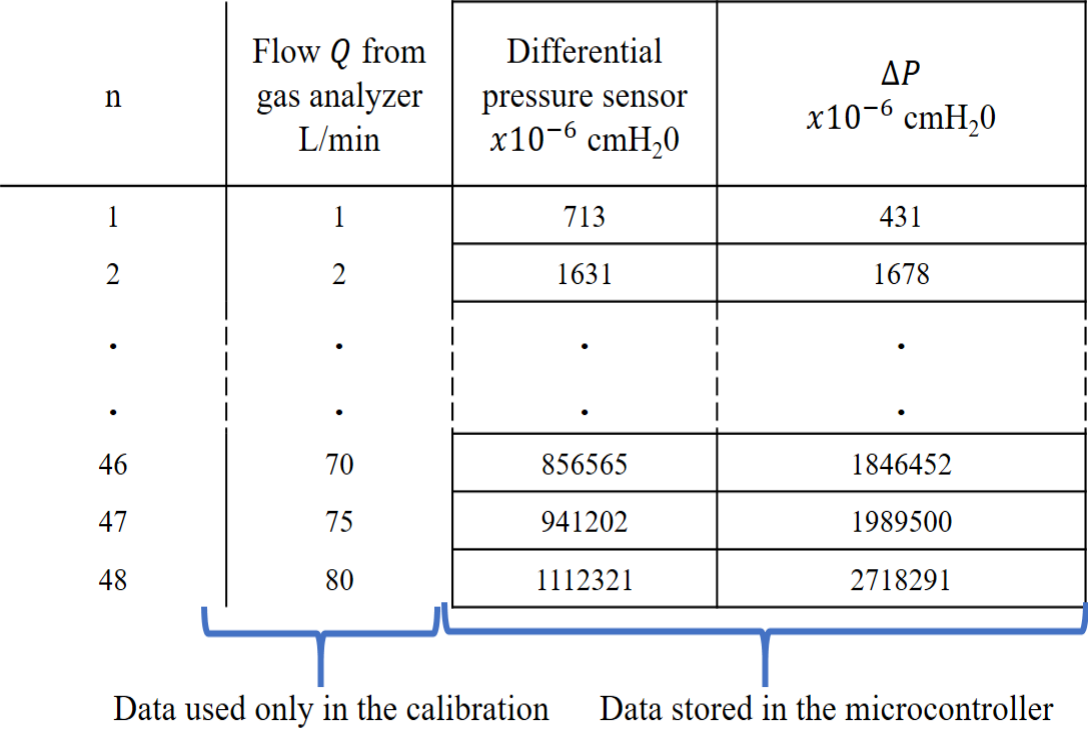
Internal array in the microcontroller created during the calibration process.

The calibration process ends when the calculation of values in the array of two columns with $n$ rows (see Fig. 4) is complete. The microcontroller stores the array to use later in the flow estimation stage. The value of $n$ corresponds to the flow value supplied. In our case, due to the sensor range, we only capture values up to 80 L/min-or the sensor saturation point, which is $n= 48$ for our system. If there is a higher-range DPS, then the calibration process can continue in steps of 5 L/min up to the sensor saturation point or the flow range to be measured. It is possible to propose a piece-by-piece regression model instead of storing the calibration values. However, the main advantage of storing data is that it is unnecessary to implement the mathematical regression in the embedded system, which can produce an additional error in the $k$ factor estimation.

The value in column one of the array (see Fig. 4) in the microcontroller is the differential pressure value read with sensor 2 (see Fig. 1). Fig. 4 shows how the information structure looks internally in the microcontroller array. The value in column two of the array is the estimated $\Delta P$ value obtained by applying (2). To estimate the density $\rho $ of the gas mixture during calibration, we proposed using only air, in accordance with the following equation:

\begin{equation*} \rho =\frac {P_{\text {abs}} \left ({\frac {\%V_{\text {Air}} m_{\text {Air}} + \%V_{O_{2}} m_{O_{2}}}{100} }\right)}{R ~T}. \tag{3}\end{equation*}

However, to calculate density when the system is operating in mechanical ventilation with a patient, it is necessary to consider the mixture of air plus oxygen, incorporating all the parameters in Table I. We recommend to carry out the calibration once a year as the offset stability of the DPS is of 0.01 Pa/year, and it is of 1 mbar/year for the atmospheric pressure sensor. According to the generic procedure for mechanical ventilators, the FiO_2_ cell must be calibrated each time that is replaced, and every time abnormal variation is observed, for example, obtaining a value greater than 100%.

**TABLE I:** Units for Estimation of the Gas Mixture Density

Symbol	Description	Unit	Value
$P_{abs}$	Absolute pressure	cmH_2_O	From atmospheric & relative pressure sensors
% $V_{Air}$	Percentage of volumetric air flow	%	From FiO_2_ cell
% $V_{O_{2}}$	Percentage of volumetric oxygen flow	%	From FiO_2_ cell
$m_{Air}$	Air molar mass	kg/mol	29
$m_{O_{2}}$	Oxygen molar mass	kg/mol	32
$R$	Reynolds number	J/mol K	8.314
$T$	Temperature	K	From temperature sensor
$\rho $	Gas mixture density	kg/ $\text{m}^{3}$	From Eq. 3

### C. Estimation Flow and Volume Process

**Fig. 5.**
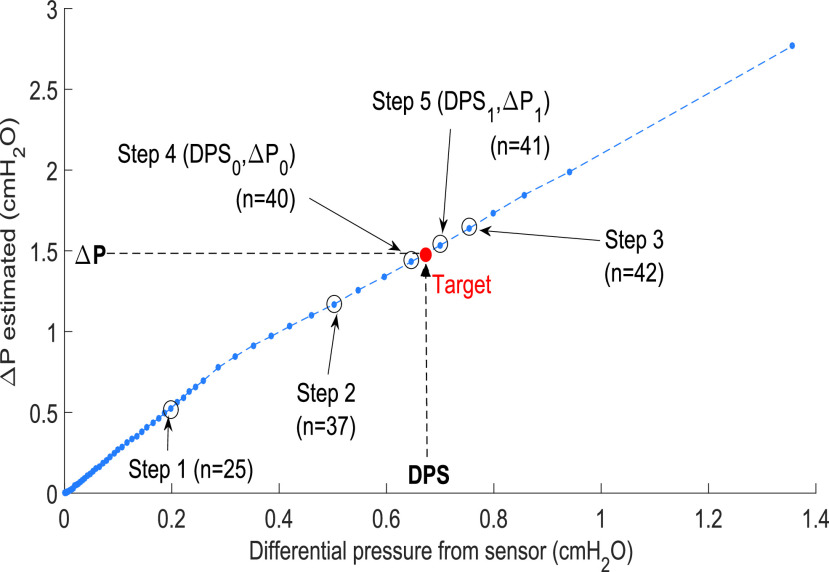
Application of the five-step O(5) dichotomous search algorithm to estimate the differential pressure value ( $\Delta P$) for DPS measurements using the data array stored in the microcontroller.

During mechanical ventilation, our system (see Fig. 2) uses (1) assuming $C_{\mathrm{ d}}= 0.7$, $A_{\mathrm{ c}}= 58$ mm^2^, and calculating the density of gas mixture according to (3). The microcontroller uses the data from the array stored (see Fig. 4) and the value read with the DPS to find the $\Delta P$ value to apply in (1). The microcontroller searches the DPS position in the array (see Fig. 5). To do this, instead of using a regression equation as other authors do [36], we applied a dichotomous search algorithm. The function in Fig. 5 shows on the $Y$-axis the estimated $\Delta P$ value that must be entered into (1) to calculate the flow correctly; the $X$-axis shows the DPS. Fig. 5 shows the advantage of this novel method. It must be remembered that we considered $C_{\mathrm{ d}}$ with a constant value. However, it is a variable function with a differential measurement of flow [27]. So, the change in gradient near $n =31$ shows the more significant intrinsic fit on the slope between each pair of sampled pressure points.

Fig. 5 shows an example of the dichotomous search algorithm working within the array of data ordered and stored in the microcontroller to determine first the x-position of the target (measured DPS value). The goal is to obtain DPS_0_ and DPS_1_, which are the differential pressures stored in the microcontroller array in column 1 (see Fig. 4). DPS_0_ and DPS_1_ are the upper and lower row in relation to the measured DPS value, and they are used for a linear interpolation using the measured DPS value to obtain $\Delta P_{0}$. In the first step, the algorithm compares the measured DPS value with the stored value corresponding to the midpoint of the array ( $n=25$, see Fig. 4), and looks for where DPS value fits; the algorithm disregards the half of the array in which the value cannot exist. In the second step, the algorithm compares the measured DPS value with the stored value corresponding in the midpoint of the remaining subarray ( $n=37$, begins at 25 and ends at 48), and again it looks for where the DPS value fits; the algorithm disregards the half of the subarray in which the value cannot exist. This action is repeated for steps 3 and 4. In step 5, the algorithm knows that the target is between $n=40$ and $n=41$, so, DPS_0_ and DPS_1_ are defined here. In the worst case scenario, the binary search is computed in a logarithmic time, performing log2 + 1 iterations. In our case, the maximum number of steps to find the value is six (for example, if the target is in $n=38$ in Fig. 5, there are two more steps necessary in reference to step 4). Once the positions of the rows between which the values have been found, a linear interpolation is performed between the data to find the value of $\Delta P$ to be entered into the next equation

\begin{equation*} \Delta P= \Delta P_{0} + \frac {\text {DPS}-\text {DPS}_{0}}{\text {DPS}_{1}-\text {DPS}_{0}}\left ({\Delta P_{1} - \Delta P_{0} }\right) \tag{4}\end{equation*}

where DPS is the differential pressure measured with the sensor. DPS_0_ and DPS_1_ are the differential pressures stored in the upper and lower row in the microcontroller array in column 1 (see Fig. 4) between the measured DPS value. $\Delta P_{0}$ y $\Delta P_{1}$ are the pressures stored in the upper and lower row in the microcontroller in column 2. We achieved the local error type (in the final step before arriving at target value) due to this interpolation process, it means that the estimation of $\Delta P$ is only a function of the variables in (4). The flow value is integrated numerically, employing the trapezoidal integral to perform the volume calculation. The equation for volume is as follows:

\begin{equation*} V_{k}=V_{k-1}+\frac {\Delta t (Q_{k} + Q_{k-1})}{2} \tag{5}\end{equation*}

where $k$ is the sample number, $\Delta t$ is the sampling time, $Q_{k}$ is the estimated flow value, and $V_{k}$ is the estimated volume in the iteration.

### D. Timing Description

To validate mechanical ventilators, it is necessary to approve a testing protocol using flow and pressure sensors with a 10%–90% rise time of ≤ 10 ms [33]. The proposed sensors in our system for relative and differential pressure have a time response ≤ 3 ms (see Section III-A) and the dichotomous search algorithm takes $10.28~\mu \text{s}$ (with six steps to find the $\Delta P$ value) in the microcontroller, which runs at 80 MHz. The flow and volume estimation process is carried out every 5 ms.

## IV. Experimental Results

To demonstrate the functionality and capabilities of our novel flow estimation method, we carried out four tests. The first test compared the estimated volume in a volume-controlled mode range from 100 up to 800 mL; this range was the minimum required for ventilators during the pandemic [33]. The second test was to validate the ability of the method to compensate for ambient conditions. The third test was to quantify the error as a function of flow, and finally, the last test was the validation of the method with different mixtures of air and oxygen. These four tests demonstrated that the method is practical, robust, and directly implementable in mechanical ventilation for patients in an ICU.

### A. Volume Estimation Test

In the first test, we considered only air at atmospheric pressure of 81 800 Pa in the gas mixture. The ventilation mode was volume-control; in this mode, the controlled variable is the flow. For the test, the circuit had a PEEP of 5-cm H_2_O, an I:E ratio (inspiratory:expiratory time ratio) of 1:1, and a frequency of 20 breaths per minute (BPM). The test consisted of capturing the volume values obtained at intervals of approximately 50 mL, starting at 100 up to 800 mL. Due to the volumes and respiratory rate, flows were evaluated in a range of 4.56–30 L/min. The metric was the absolute relative error, which is used as a measure of precision. That is, the ratio of the absolute error of a measurement with a certified instrument to the measurement being taken. The measurement instrument used as a reference was the Fluke VT900 Gas Analyzer, which has an inspiratory tidal volume accuracy of ±1.75%. It is important to mention that the most crucial validation related to flow is the volume measurement since the allowed error criteria are established in volume [33]. Of course, if the volume error exists, then there is an error in the flow estimation since the volume is the numerical integration of the flow.

**Fig. 6.**
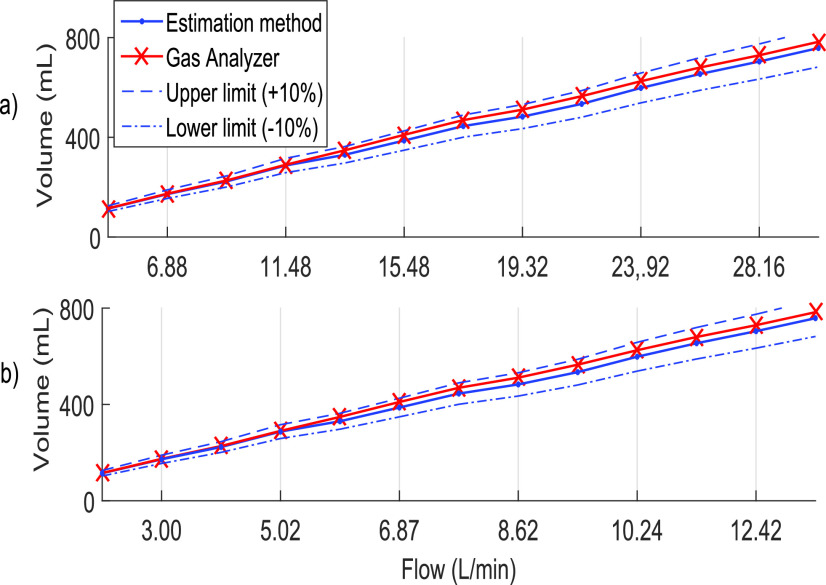
Flow measurement comparison between the proposed method and the gas analyzer. Considering as PEEP value = 5 cm H_2_O, two lungs of 600 mL with compliance of 3000 mL/Pa and resistance of 500 Pa/L/s. A height of 81 800 Pa. (a) Comparison at a respiratory rate of 20 BPM. (b) Comparison at a respiratory rate of 10 BPM.

Fig. 6 shows the results obtained in the test. Table II shows that the maximum absolute relative error for these conditions was 5.94%, giving an average relative error of 3.74%, remaining within the allowed margin. Fig. 6(b) shows the results at a frequency of 10 BPM; the reduction at this frequency directly impacts the flow, resulting in an average flow value in the range of 2.07–13.5 L/min. For both tests, Table II shows that the maximum relative error never exceeded 10%, maintaining an average relative error less than 5%.

**TABLE II:** Summary Results of Volume Estimation Comparison Test

Metric	Test a	Test b
Absolute average relative error (%)	3.74	4.01
Absolute maximum relative error (%)	5.94	6.28
Absolute minimum relative error (%)	0.88	0.72

### B. Test at Different Ambient Conditions

**Fig. 7.**
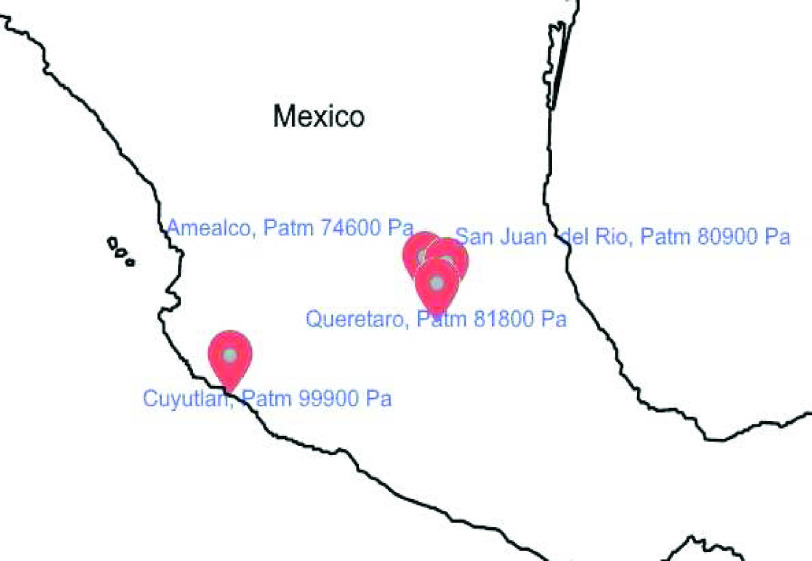
Geographic location of test sites.

We used only air and different ambient conditions (atmospheric pressure and temperature) for the second test. The ventilation mode was also volume-control. For the test, we used a PEEP of 5-cm H_2_O, an I:E ratio of 1:1, and a frequency of 20 and 10 BPM. The test was to capture the volume values obtained in steps of approximately 50 mL, starting at 100 mL and reaching 800 mL. For the test, we displaced the entire system at four different sites within Mexico. These sites were Amealco, San Juan del Rio, and Queretaro, in the Queretaro state, and at sea level, the site was in Cuyutlan, in the state of Colima (see Fig. 7). Table III shows the results obtained from the tests, showing that in all cases, the error did not exceed 10%, in addition to maintaining an average relative error lower than 5%. It is important to mention that the sea level test was carried out using the PF-300 (IMT Analytics, Switzerland) gas analyzer. Therefore, it is considered that the maximum relative error obtained in this test has reached a value of 7.26%.

**TABLE III:** Summary Results of Different Ambient Conditions Test

Metric	Amealco	SJdRio	Qro	Cuyutlan ^1^
Atmospheric pressure (Pa)	74600	80900	81800	99900
Temperature ( $^{o}\text{C}$)	27	30	26	33
Average relative error (%)	3.21	2.45	3.87	4.86
Maximum relative error (%)	6.81	4.88	6.28	7.26
Minimum relative error (%)	0.00	0.00	0.72	1.37
Standard deviation (%)	1.91	1.52	1.92	1.40

^1^ Test performed with a different gas analyzer.

### C. Error Test Based on Flow

In the third test, we considered only air at atmospheric pressure of 81 800 Pa. The ventilation mode was volume-control. For the test, the circuit had a PEEP of 5-cm H_2_O. The test consisted of varying the respiratory rate (BPM), the I:E ratio, and the tidal volume to obtain a flow value considered constant that was controlled throughout the ventilatory cycle. This test compared the dichotomous flow algorithm method with the PF-300 gas analyzer measurement and with the regression model in D-lite with only one $k$-value [35].

**Fig. 8.**
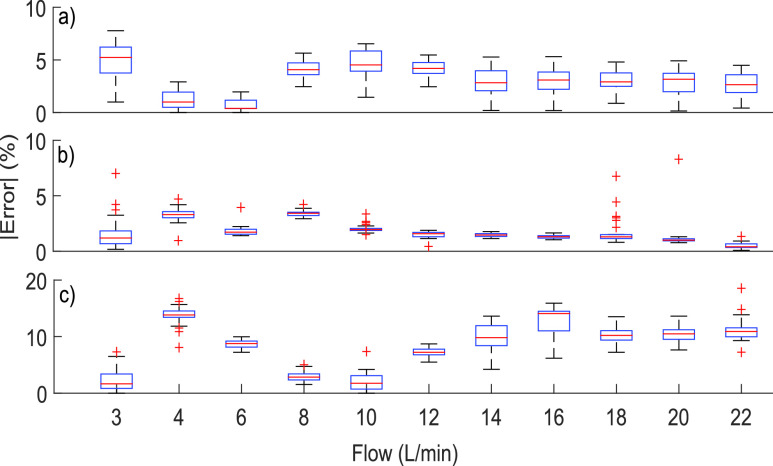
Boxplot comparing the absolute relative error of volume and the flow controlled value, taking 30 measurements per condition, from 3 up to 22 L/min. The crosses represent outlier-type values according to the boxplot construction procedure. (a) Proposed method with dichotomous search algorithm. (b) PF-300 gas analyzer. (c) Regression model in D-lite with only one $k$ value [35].

We captured 30 samples for each one flow value from 3 to 22 L/min. Fig. 8 shows the results obtained in the tests, showing that the error did not exceed 10%, in addition to maintaining a low average relative error, even for low-flow values. In addition, Fig. 8(a) shows that the variability of the proposed method was more significant than the PF-300 gas analyzer. However, the proposed method is suitable because the corresponding box plots did not show outliers (represented by a cross). There is an oscillating error between 3 and 8 L/min. However, this oscillating error arises from the commercial equipment and the method of regression too. This oscillation is related to the mechanical system used in the test. Table IV resumes the results obtained from the test, showing the good average relative error and the standard deviation of the proposed method.

**TABLE IV:** Summary Results of Error Test Based on Flow

Metric	Proposed method	PF-300	Regression model [35]
Average relative error (%)	3.08	1.79	8.33
Maximum relative error (%)	7.77	8.29	23.11
Minimum relative error (%)	0.00	0.07	0.00
Standard deviation (%)	1.70	1.11	4.48

The important thing to note about the test is that despite having an error in low-flow, this error remains within the acceptable range, showing that it is possible to use a ventilator for low-volume ventilation with this method. In addition, low-flow measurement with this method allows ventilators to activate ventilatory assistance, also known as inhalation patient flow.

### D. Volume Test With Air Plus Oxygen Mixture

**Fig. 9.**
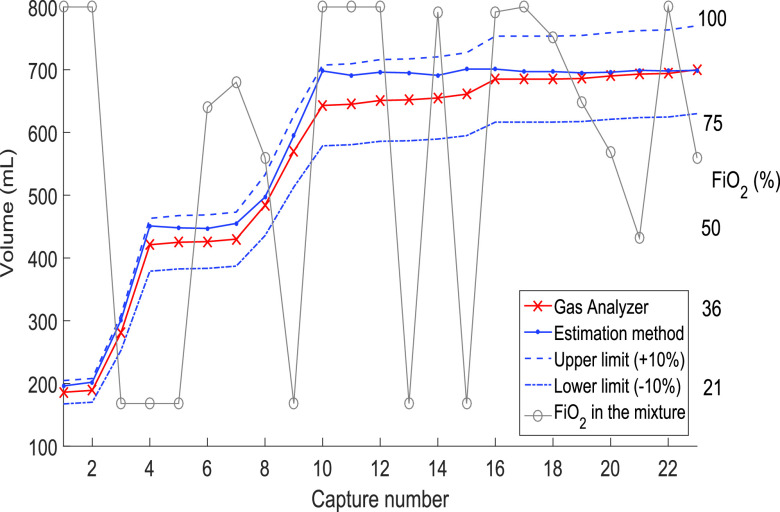
Volume measurement comparison between the proposed method and the gas analyzer varying the oxygen in the gas mixture. Considering as PEEP value = 5 cm H_2_0, two lungs of 600 mL with compliance of 3000 mL/Pa and resistance of 500 Pa/L/s. A height of 81 800 Pa, and respiratory rate of 20 BPM.

For the last test, we varied the percentage of FiO_2_ in the gas mixture. The atmospheric pressure was 81 800 Pa. The mode of ventilation was the volume control. For the test, a PEEP of 5-cm H_2_O, an I:E ratio of 1:1, and a frequency of 20 BPM were used. The test consisted of varying the FiO_2_ values at random and increasing the volume up to 800 mL. Fig. 9 shows the results obtained when comparing the volume measurement with the Fluke VT900 Gas Analyzer. Fig. 9 shows that the estimated volume (numerical integration of the flow) works correctly despite immediate variations and fluctuations in the percentage of FiO_2_ in the mixture. The samples for this test were few due to the shortage of oxygen. However, during the development of the method and already in the application, multiple tests were carried out, and the measurement was always kept within the 10% margin.

## V. Discussion

Our novel method decreased the error in low flow concerning previous works since the regression model considers a global error. In contrast, our method, through the dichotomous search, focuses on the local error. The primary consideration in our proposed method is that the error is concerning the accuracy in the measurement of the flow values (in the calibration stage) higher and lower than the sought value. Fig. 8 presented an interesting result since, for the low flow values of 4 and 6 L/min, the error obtained was lower than the others. This improvement in the estimation is related to the airflow way and calibration process. It is important to remember that the air source used for the test was a ventilator AMBU-based prototype. The flow (obtained varying BPM, I:E ratio, and volume) supplied was more stable at those values; therefore the 5 ms sampling achieved a better numerical integration. The calibration stage also contributes toward the reduced error because the error is local. So, if the process was better for those points (for example, with more stable conditions), it improved the estimation.

Once the potential and advantages of this novel method have been demonstrated through the four tests mentioned in Section IV, two essential points to consider for all developers should be discussed. The first point to consider is the impact on the flow estimate of the error in each model parameter. The second point to be discussed is the impact on the measurement if it is not considered that the mixture is air plus oxygen.

All flow and volume computations are carried out with a constant sampling time of 5 ms, with the microcontroller operating at 80 MHz. To correctly calculate flow according to (1), the algorithm must consider the absolute pressure. It implies that every 5 ms, the density in the mixture should be calculated by adding together the atmospheric pressure and airway pressure. Although this point-to-point calculation would improve the estimation of the flow value, it is also true that it consumes more computing time. Depending on the type of application required, the developer must decide whether to consider airway pressure in the density computation or not. If all the computation of a ventilator is programmed in the same microprocessor, the density estimation can be done for each ventilatory cycle using a single constant value of density for all the points within the ventilatory cycle.

**Fig. 10.**
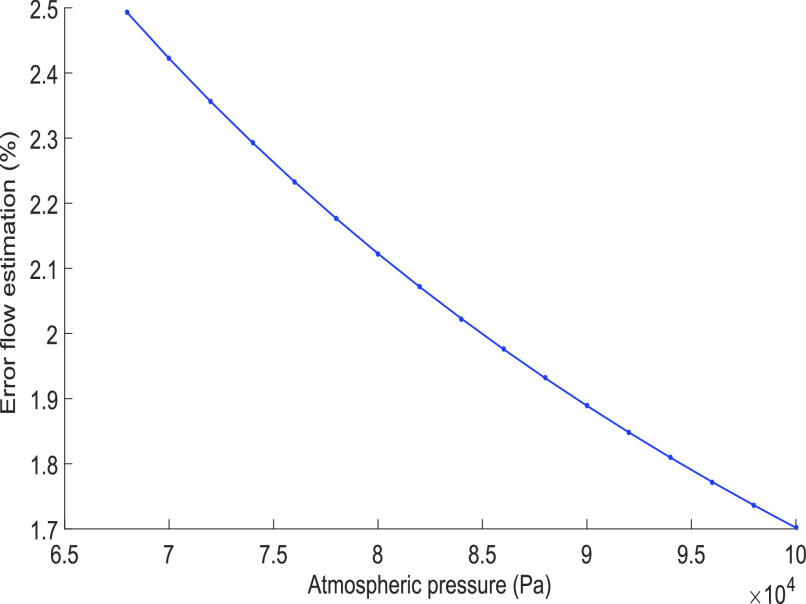
Error in flow estimation concerning the atmospheric pressure variation (omitting pressure in the airway) for calculating the density of the mixture.

The question to be considered is the impact on the measurement error that this calculation may have. This method facilitates the operation which is performed every 5 ms by reducing the number of arithmetic operations performed by the microcontroller. Fig. 10 shows the impact on the flow measurement if the density of the mixture is adjusted at the beginning of each respiratory cycle considering only the atmospheric pressure. The result indicates that the error in flow caused by this technical consideration generates an error of between 1.7% and 2.4%. The impact of this error can be seen in Fig. 6, where the non-consideration of the relative pressure (pressure in the airway every 5 ms) generated that the flow estimation with this method applied in a microcontroller is always below the value indicated by the gas analyzer.

**Fig. 11.**
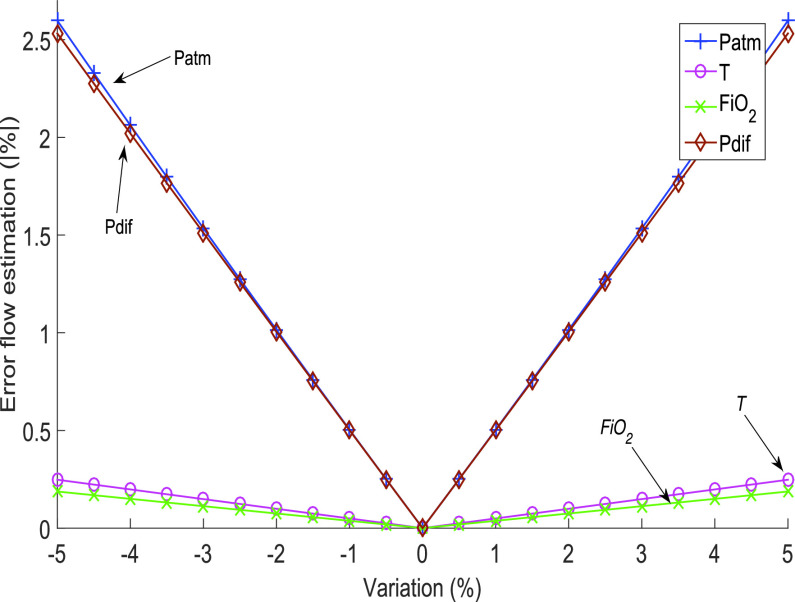
Error in the estimation of flow as a function of the variation of each parameter concerning its real value. The analysis looks at the method that does not consider pressure in the airway to calculate the density of the mixture.

Since the analysis considering the relative pressure for the density estimation was interesting to determine its impact on the flow estimation, this analysis was extended to all variables considered in this method. Fig. 11 shows the flow estimation error as a function of the variation of each parameter, which was simulated relative to their true value. This analysis shows that varying the atmospheric and relative pressures generates the most significant impact.

**Fig. 12.**
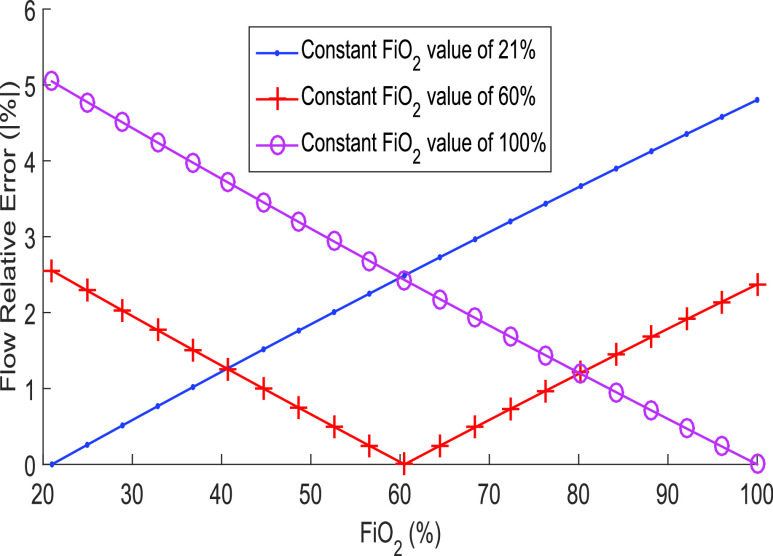
Error in the flow estimation considering no FiO_2_ cell available to measure the percentage of oxygen in the mixture and using a constant value of 21%, 60%, and 100% for the density estimation.

The second point to be discussed is related to the flow estimation error as a result of not considering the percentage of FiO_2_ when performing gas density calculations and only considering the density of the air. Fig. 12 shows that if developers do not have a FiO_2_ cell to measure the percentage of oxygen during ventilation, it is better to consider a constant value of 60% to estimate the density of the gas mixture. Using 60% of FiO_2_ will cause an absolute error maximum of 3% in all the range of FiO_2_ of 21 up to 100%. This is not the case if only air or 100% oxygen is considered in density calculation. In both cases, an error greater than 5% is reached, which is an error value risk since in this novel method, errors of 6% are already reached.

In general, our method improves the use capabilities of many ventilators since the medical team widely uses flow and volume to determine patient progress or clinical status [10]. To implement this new method, the developers need to have a gas analyzer; this condition may seem difficult to achieve; however, each ventilator delivered to a hospital must comply with the volume within the established 10% margin. The user carries out this validation only with a calibrated gas analyzer; then, if a ventilator does not comply with the established volume margin, the developer can use this method and be sure that it will accurately measure flow regardless of changes in ambient conditions.

Finally, it should be noted that other authors [27], [29] reported a maximum error between 4% and 5%, when accurately considering all the model variables or applying several regression models. Others [35] have used a single $k$ value for the entire flow range, nevertheless presenting a substantial error for low flow estimation. Our novel method presents various advantages compared to previous methods and an average relative error of up to 4.86%.

## VI. Conclusion

The COVID-19 pandemic demonstrated the capacity and willingness to support on the part of scientists to develop solutions that would help mitigate, reduce or treat the effects caused by the virus. One of these solutions was developing mechanical ventilators, where multiple and ingenious solutions have been presented. State of the art research shows that the ability to estimate proximal flow to the patient is vital and that many developed ventilators require a robust and cheap measurement system for this variable. We presented and validated a novel method for flow estimation, where our novel approach adapted the dichotomous search algorithm instead of using a regression model for the D-lite sensor. The dichotomous search algorithm allowed us to find a differential pressure value to be entered into the flow estimation equation (1). The binary search algorithm was applied to a dataset stored in the microcontroller, the dataset is a function of the differential pressure values read by the sensor and the estimated values obtained in the calibration stage.

The main advantages of this method are as follows.

1) Intrinsic adjustment of a $k$ value for each differential pressure value, instead of a global fixed $k$ value obtained by a linearized regression.
2) A good low flow estimation, useful as a trigger during assistant ventilatory cycles.
3) Determination of the calibration and measurement process.
4) Viability of implementation in low-cost microcontrollers, as it does not require significant computing operations every 5 ms.
5) Flow estimation considering the value of FiO_2_ in the mixture, which makes it directly applicable under real mechanical ventilator conditions.

We implemented this novel method in our mechanical ventilators, and the measurement has been validated for Vti and Vte with different conditions using the volume estimation test (A) and the volume test with air plus oxygen mixture (D) during the development process, final inspection in the manufacturing process with external and auditable procedures, and during delivery of ventilators in hospitals, always complying with the established volume error criteria.
